# Oxytocin Protects PC12 Cells Against β-Amyloid-Induced Cell Injury

**DOI:** 10.3390/ph18030390

**Published:** 2025-03-10

**Authors:** Mohammed Mufadhe Alanazi, Awatif B. Albaker, Lamia A. Alzaagi, Jawza F. Alsabhan, Fawaz Alasmari, Mohammed M. Almutairi, Metab S. Alharbi, Abdullah F. Alasmari, Faleh Alqahtani, Sary Alsanea

**Affiliations:** 1 Department of Pharmacology and Toxicology, College of Pharmacy, King Saud University, Riyadh 11451, Saudi Arabia; 2 Department of Clinical Pharmacy, College of Pharmacy, King Saud University, Riyadh 11451, Saudi Arabia

**Keywords:** Alzheimer’s disease, oxytocin, PC12 cells, β-amyloid protein, Bcl-2, BAX, ERK1/2

## Abstract

**Background/Objectives**: Neurodegenerative diseases, particularly Alzheimer’s disease (AD), are characterized by progressive cognitive decline and non-cognitive symptoms that significantly affect health and quality of life. Beta-amyloid (Aβ) protein accumulation is a key factor in AD pathology, leading to neuronal damage. Oxytocin (OXT), a neuropeptide with neuroprotective potential, has garnered interest owing to its ability to mitigate neurotoxicity. We hypothesized that oxytocin could protect PC12 cells from Aβ-induced cytotoxicity through antioxidant effects and modulation of apoptotic pathways (i.e., mitochondrial and MAPK pathways). In this study, we aim to assess oxytocin’s protective effects on cell viability, oxidative stress, mitochondrial function, and apoptotic signaling. **Methods**: PC12 cells were treated with Aβ25–35 and pre-treated with varying oxytocin concentrations to assess cell viability, reactive oxygen species (ROS) generation, and mitochondrial membrane potential. Western blotting was performed to analyze the effects on mitochondrial apoptosis and MAPK pathways. **Results**: Oxytocin treatment significantly improved cell viability in a dose-dependent manner and reduced Aβ-induced oxidative stress and mitochondrial dysfunction. Oxytocin-treated groups exhibited decreased ROS levels, increased mitochondrial membrane potential, and modulation of apoptosis-related proteins. Oxytocin upregulated phosphorylated ERK1/2 and Bcl-2 while downregulating BAX and caspase-3, reducing the BAX/Bcl-2 ratio. **Conclusions**: Oxytocin effectively protects PC12 cells from Aβ-induced neurotoxicity, highlighting its potential as a therapeutic agent for AD. Further research is needed to clarify oxytocin’s mechanisms and clinical implications in AD treatment.

## 1. Introduction

Neurodegenerative diseases such as Alzheimer’s disease (AD) are age-dependent disorders that significantly affect human health [1]. They are characterized by progressive cognitive impairment that can lead to debilitating non-cognitive symptoms, such as sleep and appetite disturbances, and neuropsychiatric alterations, including depression and apathy [2]. The causes of AD are not fully understood; however, evidence points to amyloid plaques and neurofibrillary tangles as contributing factors. Amyloid plaques consist of Aβ protein derived from an origin protein called the amyloid precursor protein (APP), which has four parts—α, β, γ, and η—that create three pathways. C-terminal fragment (CTF)-α is helpful for nerve cells, CTF-β may harm nerve cells, and η-secretase produces insoluble Aβ proteins, which accumulate in the brain and lead to neurotoxicity and cell death [3,4,5].

B-amyloid (Aβ) and its amino acid sequence were first reported in 1984 and labeled as a primary constituent of meningovascular polymorphic deposits in patients with Down syndrome [6]. The full-length Aβ protein contains 40 or 42 amino acids, whereas Aβ25-35, also known as a toxic fragment of the Aβ protein, is the shorter fragment used as a model system to study Aβ aggregation and its role in AD [7,8].

Numerous studies have demonstrated a clear relationship between mitochondrial dysfunction and AD pathogenesis [9,10]. Notably, Aβ significantly induced neuronal death in cultured neurons as well as in the brains of treated wild-type mice through upregulating apoptotic genes, including caspase-3 [11]. In fact, caspases contribute to two different apoptotic pathways, including the extrinsic pathway, which is triggered by death receptors outside the cell, and the intrinsic pathway, which is initiated by mitochondrial membrane depolarization due to cellular injury. Both pathways involve caspases [12]. Notably, the Bcl-2 family, comprising 25 genes, controls apoptotic mitochondrial events. Some proteins promote cell survival (e.g., Bcl-2, Bcl-x, Bcl-XS, Bcl-XL, Bcl-w, and BAG), whereas the others promote cell death (e.g., Bcl-10, BAX, Bak, Bid, Bad, Bim, Bik, and Blk). The enzymatic activity of the mitochondrial respiratory chain complex (RCC) is essential for two reasons. First, it generates the mitochondrial inner membrane potential (mtΔΨ), reflecting the health of the mitochondria and cells [13]. Second, electron leakage from the RCC leads to the formation of reactive oxygen species (ROS) [14].

Several studies have indicated that Aβ disrupts mitochondrial redox activity and increases ROS generation, leading to neuronal death [15,16,17]. Additionally, the accumulation of unfolded proteins in the endoplasmic reticulum (ER) induces ER stress, which is associated with neurodegenerative disorders, such as AD, Parkinson’s disease, and polyglutamine diseases [18,19].

Recent studies have highlighted a strong connection between dysregulation in extracellular regulated kinases (ERK), Bcl-2 expression, Aβ, and cognitive dysfunction. It has been shown that activation of ERK1/2 expression can protect against Aβ-induced apoptosis [20]. Furthermore, Aβ has been shown to downregulate the anti-apoptotic Bcl-2 protein and decrease the expression of the pro-apoptotic BAX protein, which contributes directly to inducing the apoptosis process [21]. Collectively, these findings can contribute to cognitive decline that is associated with AD [22,23].

Recently, research attention has shifted to investigating and targeting the pathways responsible for Aβ accumulation in the brain tissue, particularly oxidative stress. Consequently, antioxidants have become one of the promising therapeutic approaches for AD. In this context, oxytocin (OXT), which is a neuropeptide, has antioxidant effects that may benefit a range of neurodevelopmental and neurodegenerative diseases. OXT is synthesized in the hypothalamus and released from the neurohypophysis and nerve terminals in response to physiological stimuli. Its main function is mediated by the widespread oxytocin receptors (OXTRs) throughout the brain. OXT has been implicated in several physiological functions, ranging from reproduction to social behaviors and non-social behaviors [24]. In addition, OXT has been reported to exert protective effects against neurotoxicity induced by various stressors [24]. OXT has been shown to have a protective effect on various body systems. Recent studies have revealed that OXT protects the cardiovascular system against various injuries, apoptosis, and inflammation. It also slows down the progression of atherosclerosis and promotes cardiovascular recovery [25,26,27]. Therefore, we hypothesized that OXT could protect neuronal cells from Aβ-induced cytotoxicity through antioxidant effects and modulation of apoptotic pathways (i.e., mitochondrial and mitogen-activated protein kinase [MAPK] pathways). To test this hypothesis, we used PC12 cells to assess the protective effects of OXT on cell viability, oxidative stress, mitochondrial function, and apoptotic signaling pathways.

## 2. Results

### 2.1. Dose–Response Curves of Amyloid Beta (Aβ) and Oxytocin (OXT) on PC12 Cells

**Figure 1:**
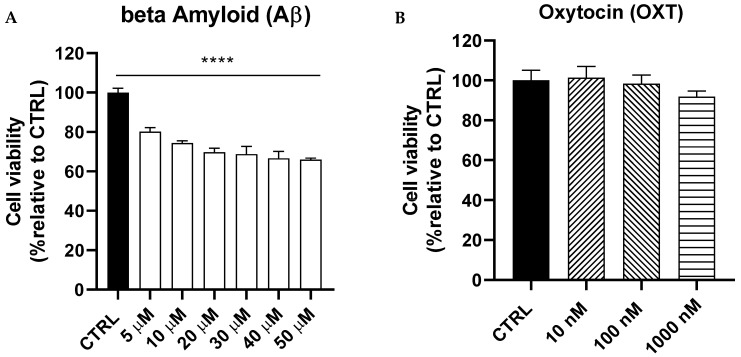
Dose–response curve of beta-amyloid (Aβ) (**A**) and oxytocin (OXT) (**B**). Cells were treated with either Aβ (5, 10, 20, 30, 40, and 50 μM) for 48 h or OXT (10, 100, 1000 nM) for 2 h. Data normalized to the control group and expressed as Mean ± SEM. **** *p* < 0.0001 compared to control group.

We conducted a dose–response curve for Aβ (5–50 µM) over 48 h to determine the optimal concentration for subsequent experiments. Our findings indicated that Aβ significantly reduced the viability of PC12 cells in a dose-dependent manner (*p* < 0.0001). We selected the Aβ dose of 20 µM, which led to a viability reduction of approximately 30% for the upcoming experiments (Figure 1A). Additionally, we performed a dose–response curve for oxytocin (OXT) at concentrations of 10, 100, and 1000 nM on PC12 cells to confirm that these doses do not exert toxic effects. Our study demonstrated that the tested concentrations of OXT had no impact on the viability of PC12 cells (Figure 1B).

### 2.2. Investigating the Protective Effect of OXT Against Aβ-Induced Cytotoxicity

#### 2.2.1. OXT Protects PC12 Cells Against Aβ-Induced Cytotoxicity

**Figure 2:**
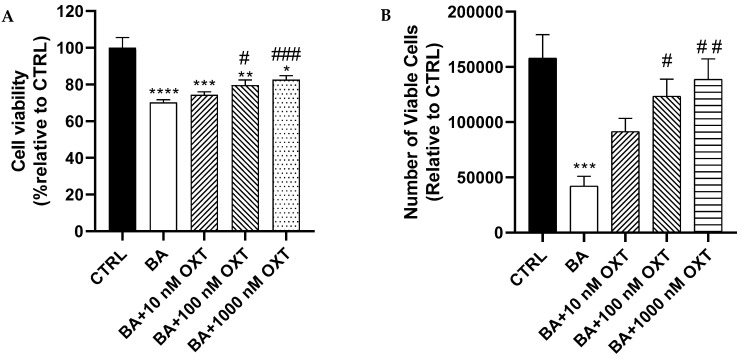
Effect of OXT in PC-12 cells vitality against Aβ induces cytotoxicity and cell proliferation using MTT assay (**A**) and Trypan blue staining assay (**B**). Cells were pre-treated with OT (10, 100, 1000 nM) for 2 h, then incubated with 20 μM Aβ for another 48 h. Data expressed as number of viable cells per mL, normalized to the control group. The result showed by % compared with the control group and Aβ group. Mean ±SEM. * *p* < 0.05, ** *p* < 0.01, *** *p* < 0.001, and **** *p* < 0.0001 compared with control group. # *p* < 0.05, ## *p* < 0.01, and ### *p* < 0.001 compared with Aβ group only.

In comparison with Group 2 (the Aβ-treated group), Groups 3–5 (pre-treated with OXT for 2 h) demonstrated a significant improvement in cell viability in a dose-dependent manner (*p* < 0.05) and (*p* < 0.001), respectively. (Figure 2A).

#### 2.2.2. OXT Protects PC12 Cells Against Aβ-Induced Cytotoxicity (Trypan Blue Staining Assay)

A significant reduction (73.6%) in cell count numbers was observed for cells in Group 2 compared with those in the control group (*p* < 0.001). In contrast, a slight improvement (43%) in cell numbers was observed in cells pre-treated with 10 nM OXT (Group 3) compared with cells in Group 2 (Figure 2B). Interestingly, Groups 4 and 5 showed a significant increase in cell numbers (*p* < 0.05 and *p* < 0.01, respectively) compared with the group treated with Aβ alone (Group 2).

#### 2.2.3. OXT Protects PC12 Cells Against Aβ-Induced Oxidative Stress

**Figure 3:**
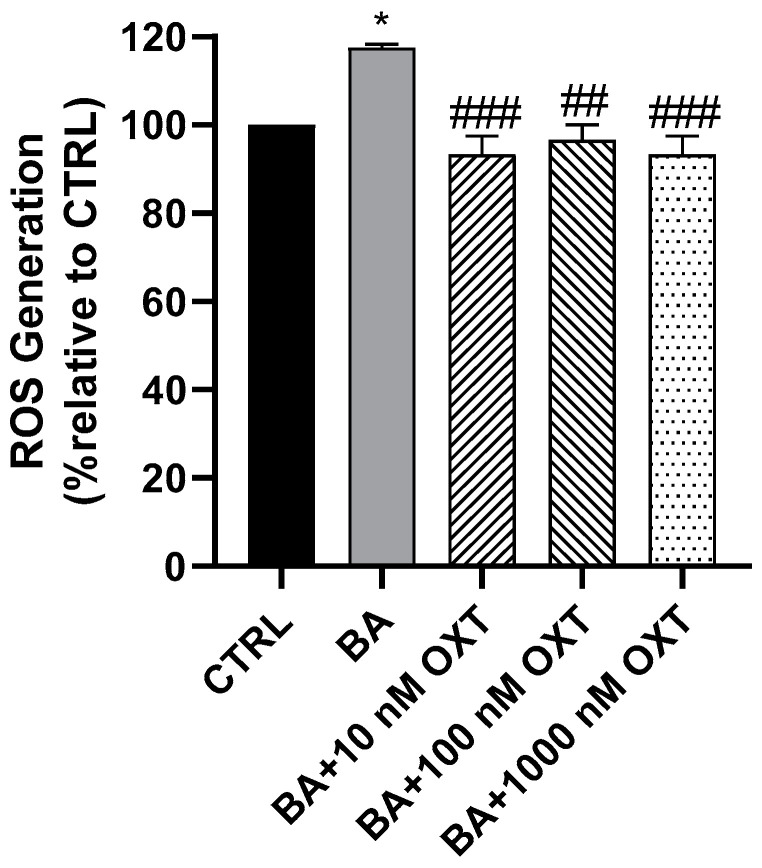
Effect of OXT on ROS production in PC12 cells against Aβ induced oxidative stress. The cells were pre-treated with OXT (10, 100, and 1000 nM) for 2 h and then treated with 20 μM Aβ incubated for 48 h. ROS production in PC12 cells was detected using the DCFDA—Cellular ROS Assay Kit. Data normalized to the control group and expressed as Mean ± SEM. * *p* < 0.05 compared with control group. ## *p* < 0.01, and ### *p* < 0.001, or compared with group incubated with Aβ only.

A 20% increase in ROS production was observed in PC12 cells in Group 2 (Figure 3) compared with those in the control group (*p* < 0.05). In addition, a significant reduction in ROS production was observed in Groups 3–5 compared with Group 2 (*p* < 0.05).

#### 2.2.4. OXT Protects PC12 Cells Against Aβ-Induced Mitochondrial Dysfunction

**Figure 4:**
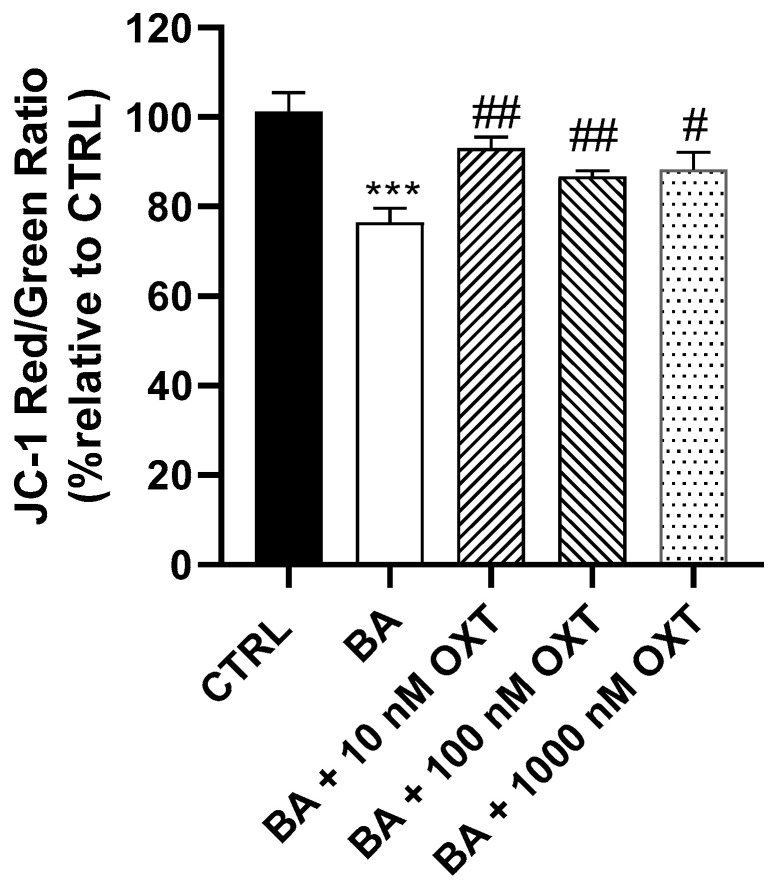
Effect of OXT against Aβ induced mitochondrial membrane dysfunction. Cells were pre-treated with OXT (10, 100, and 1000 nM) for 2 h, then treated with 20 μM Aβ incubated for 48 h to detect changes in Mitochondrial Membrane Potential using a JC-1 Assay kit. Data normalized to the control group and expressed as Mean ± SEM. *** *p* < 0.001 compared with the control group. # *p* < 0.05 and ## *p* < 0.01, or compared with the group incubated with Aβ alone.

Cells in Group 2 had a low level of red/green ratio compared with cells in Groups 1 and 3–5 (*p* < 0.05) (Figure 4). Furthermore, cells in the OXT groups (Groups 3–5) displayed a significant 24% increase in red/green ratio of (*p* < 0.01) compared with cells in Group 2. Compared with Group 2, the highest red/green ratio level was detected in Group 3, followed by Groups 5 and 4 (14%, 9%, and 7%, respectively). A decreased ratio of red/green signals indicates mitochondrial damage and cell apoptosis.

### 2.3. Investigating the Protective Effect of OXT Against Aβ-Induced Cytotoxicity Through Modulation of Mitochondrial Apoptosis and MAPK Pathways

#### 2.3.1. Effects of OXT on ERK1/2 Phosphorylation

**Figure 5:**
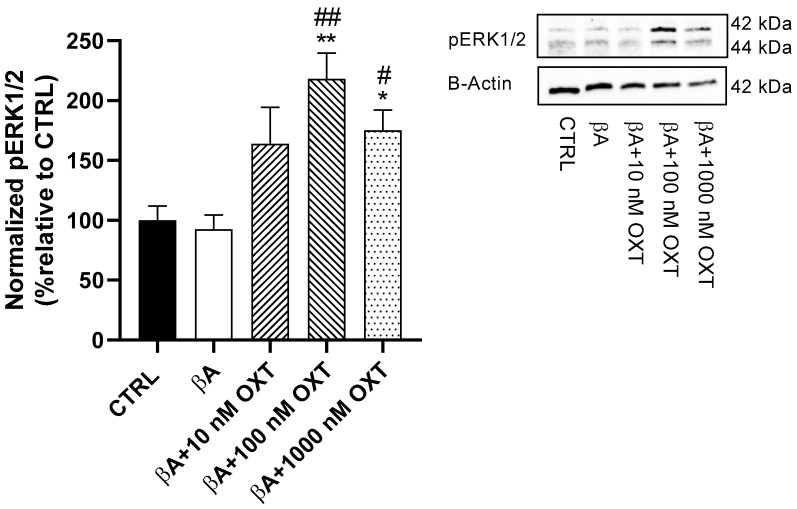
Effect of OXT on pERK1/2 expression in PC12 cells exposed to Aβ. Cells were pre-treated with OXT (10, 100, and 1000 nM) for 2 h and then treated with 20 μM Aβ for 48 h. We applied Western blotting to detect targeted protein excretion (pERK 1/2). Data normalized to the control group and expressed as Mean ± SEM * *p* < 0.05 and ** *p* < 0.01 compared with control group. # *p* < 0.05, and ## *p* < 0.01, or compared with group incubated with Aβ alone.

Our results displayed the maximum upregulation of 118% for pERK1/2 observed in Group 4 compared with Groups 1 and 2 (*p* < 0.01). In addition, Groups 3 and 5 demonstrated significant upregulations of 63% and 75%, respectively, in pERK 1/2 (*p* < 0.05). Thus, OXT in all three concentrations displayed significant upregulation of pERK1/2. In contrast, Group 2 showed no remarkable reduction in pERK1/2 compared with the control group (Figure 5).

#### 2.3.2. Effect of OXT on Bcl-2 Expression

**Figure 6:**
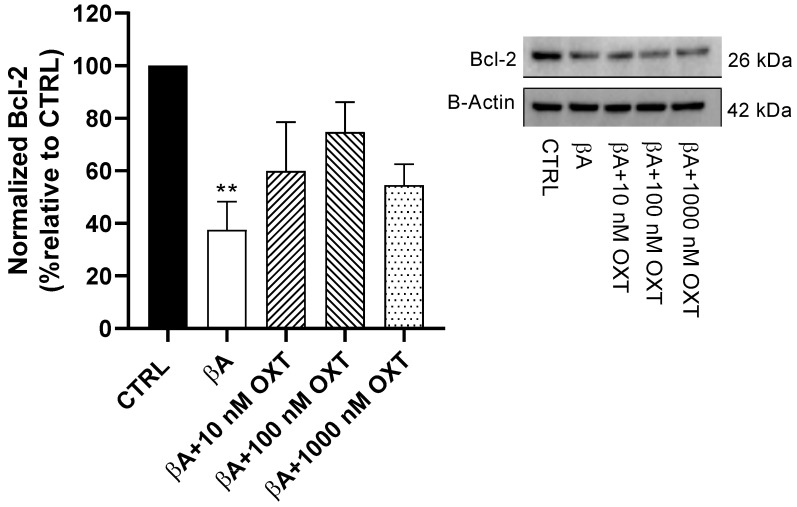
Effect of OXT on Bcl-2 expression in PC12 cells exposed to Aβ. Cells were pre-treated with OXT (10, 100, and 1000 nM) for 2 h, then treated with 20 μM Aβ for 48 h. We applied Western blotting to detect targeted protein excretion (Bcl-2). Data normalized to the control group and expressed as Mean ± SEM. ** *p* < 0.01 compared with control group.

Our WB results showed that cells in Group 2 showed a significant decrease (60%) in Bcl-2 protein expression compared with the control group (*p* < 0.01) (Figure 6). Compared with Group 2, a non-significant increase in Bcl-2 protein expression was observed by 20%, 40%, and 15% in Groups 3, 4, and 5, respectively. Notably, Group 4 demonstrated the maximum increase in Bcl-2 protein expression (Figure 6).

#### 2.3.3. Effect of OXT on BAX Expression

**Figure 7:**
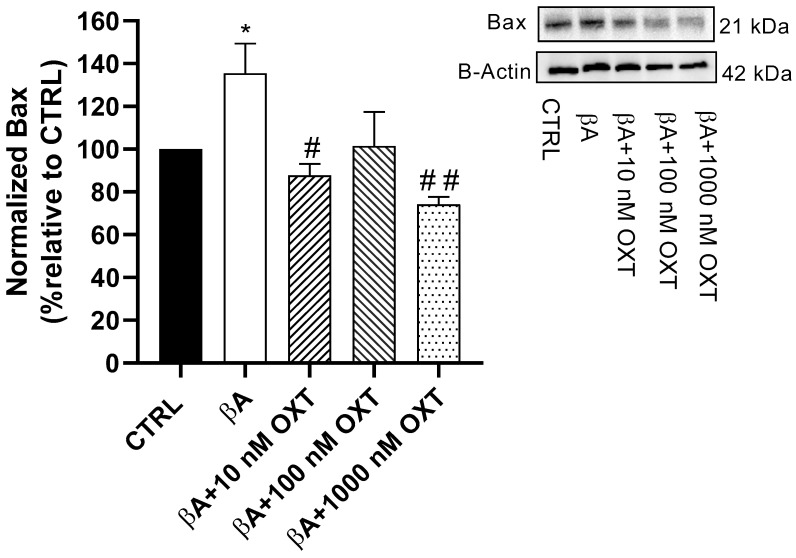
Effect of OXT on BAX expression in PC12 cells exposed to Aβ. Cells were pre-treated with OXT (10, 100, and 1000 nM) for 2 h, then treated with 20 μM Aβ25-35 for 48 h. We applied Western blotting to detect targeted protein excretion pro-apoptotic mediator BAX. Data normalized to the control group and expressed as Mean ± SEM. * *p* < 0.05 compared with control group. # *p* < 0.05 and ## *p* < 0.01, or compared with group incubated with Aβ alone.

Our results showed a significant elevation of 35.5% in BAX protein expression in Group 2 compared with the control group (*p* < 0.05) (Figure 7). Additionally, compared with Group 2, a significant reduction in BAX protein expression was observed by 47.5%, 49.5%, and 61% in Groups 3, 4, and 5, respectively (*p* < 0.05, *p* < 0.01 and *p* < 0.001). Overall, cells from Group 5 demonstrated the highest reduction compared with the other OXT concentrations.

#### 2.3.4. Effect of OXT on BAX/Bcl-2 Ratio

**Figure 8:**
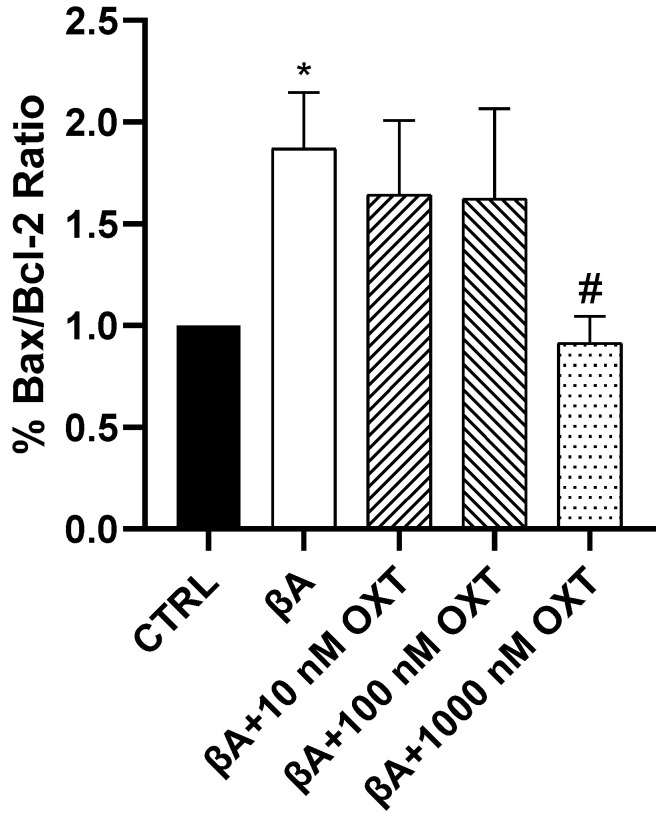
Effect of OXT on BAX/Bcl-2 ratio and in PC12 cells exposed to Aβ. Cells were pre-treated with OXT (10, 100, and 1000 nM) for 2 h, then treated with 20 μM Aβ25-35 for 48 h. We applied Western blotting to detect targeted protein excretion. Data normalized to the control group and expressed as Mean ± SEM. * *p* < 0.05 compared with control group. # *p* < 0.05 compared with group incubated with Aβ alone.

Our results showed a significant elevation of 200% in the BAX/Bcl-2 ratio in Group 2 compared with the control group (*p* < 0.05) (Figure 8). Additionally, compared with Group 2, a significant reduction in the BAX/Bcl-2 ratio of 100% was shown only in Group 5 (*p* < 0.05). The other OXT concentrations showed a trend reduction in the BAX/Bcl-2 ratio compared with Group 2. Interestingly, only Group 5 significantly mitigated the BAX/Bcl-2 ratio compared with Group 2 (*p* < 0.05) (Figure 8).

#### 2.3.5. Effect of OXT on Caspase-3 Expression

**Figure 9:**
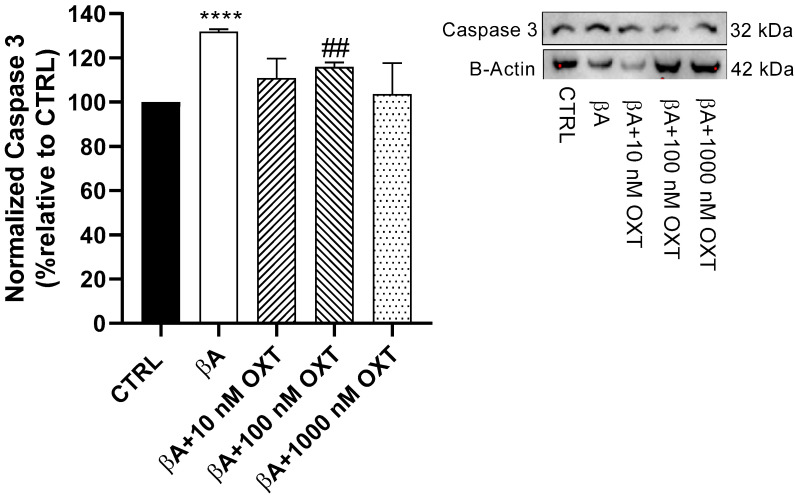
Effect of OXT on caspase-3 expression in PC12 cells exposed to Aβ. Cells were pre-treated with OXT (10, 100, and 1000 nM) for 2 h and then treated with 20 μM Aβ25-35 for 48 h. We applied Western blotting to detect targeted protein excretion pro-apoptotic mediator BAX. Data normalized to the control group and expressed as Mean ± SEM. **** *p* < 0.0001 compared with control group. ## *p* < 0.01, or compared with group incubated with Aβ alone.

Our results highlighted a significant elevation (30%) in caspase-3 protein expression in Group 2 compared with the control group (*p* < 0.0001) (Figure 9). Additionally, compared with Group 2, reduced caspase-3 protein expression by 20%, 10%, and 30% was observed in Groups 3, 4, and 5, respectively. Cells in Group 4 showed the highest reduction in caspase-3 protein expression compared with the other OXT concentrations (*p* < 0.01) (Figure 9).

## 3. Discussion

### 3.1. Investigating the Protective Effect of OXT Against Aβ-Amyloid-Induced Cytotoxicity on PC-12 Cells: Effect on Cell Proliferation and Viability

#### OXT Protects PC12 Cells Against Aβ-Induced Cytotoxicity

Our results demonstrated that OXT protects PC12 cells against Aβ-induced cytotoxicity. Aβ significantly reduced cell viability compared with the control. On the other hand, OXT-treated groups showed a significant elevation of cell viability compared with groups treated with Aβ alone (Figure 2A). According to prior findings, the effect of OXT in promoting cell viability has been confirmed in the glial cell line (U-87MG), indicating that OXT increases cell viability [28]. In rat hippocampal cells, OXT was found to enhance cell viability and proliferation, as well as facilitate the neurogenesis process. These effects were associated with improved positive emotional and social behaviors in the subjects [29]. Interestingly, Hsieh et al. have revealed that OXT induced neurite outgrowth on PC12 cells, which might indicate that OXT mediated this effect through the interaction between OXT and OXT receptors (OXTR) [30]. Our results, in conjunction with prior research, indicate that OXT has the potential to counteract the toxic effects of Aβ and safeguard cells from apoptotic processes. Furthermore, our results indicate that oxytocin (OXT) offers protective effects against the reduction in proliferation induced by amyloid-beta (Aβ) in PC12 cells. Aβ markedly decreased cell proliferation when compared with the control group. On the other hand, OXT-treated groups showed a significant elevation of cell number compared with the group treated with Aβ alone (Figure 2B). Additionally, other research has demonstrated that OXT promotes neurogenesis in the hippocampus of rats, a brain region critical for learning, memory, and emotional processing. This mechanism may contribute to the association of OXT with enhanced positive emotional and social behaviors in these animals [29]. Numerous studies have demonstrated that Alzheimer’s disease and other neurodegenerative diseases are linked to alterations in emotional and social behaviors [31,32]. Our findings suggest that OXT has the ability to counteract the toxic effects of Aβ and protect cells from cell death, highlighting its potential role in the development of novel treatments for AD. Nevertheless, additional research is necessary to validate the impact of oxytocin on the behavioral changes related to AD.

### 3.2. Investigating the Protective Effect of OXT on PC-12 Cells Against Aβ-Amyloid-Induced ROS Generation

#### OXT Protects PC12 Cells Against Aβ-Induced ROS

This study found that OXT protects PC12 cells against Aβ-induced oxidative stress. In this study, we have shown that Aβ significantly increased ROS generation compared with the control. On the other hand, OXT-treated groups showed lower ROS generation compared with the group treated with Aβ alone (Figure 3). These findings align with many studies that have proposed that oxidative stress is recognized as one of the mechanisms implicated in the pathology of AD. Interestingly, it has been demonstrated that oxidative stress can significantly contribute to Aβ aggregation, either as a consequence or as an inducer of this process [15]. Indeed, we have confirmed the antioxidant effects of OXT, which is aligned with many studies that have been reported on brain disorders [33]. Interestingly, the antioxidant effect of OXT is not limited to the brain; however, it has been shown in a variety of systems, including the cardiovascular, gastrointestinal, and hepatic systems and ischemia/reperfusion injuries of the urinary system [34,35,36]. Our findings confirmed that OXT has an antioxidant effect against Aβ-induced oxidative stress, which might provide an innovative approach to managing AD.

### 3.3. Investigating the Protective Effect of OXT on PC-12 Cells Against Aβ-Amyloid-Induced Mitochondrial Dysfunction

#### OXT Protects PC12 Cells Against Aβ-Induced Mitochondrial Membrane Disruption

Our results showed that OXT protects PC12 cells against Aβ-induced modification in mitochondrial membrane potential. We demonstrated that Aβ decreases mitochondrial membrane potential significantly compared with the control, whereas groups treated with OXT showed a significant elevation of mitochondrial membrane potential compared with those treated with Aβ alone (Figure 4). One of the most significant effects of apoptosis on mitochondria is the loss of mitochondrial transmembrane potential (ΔΨM). Mitochondria is vital in the regulation of energy production and Ca^2+^ homeostasis within cells [37]. Indeed, it has been shown that hyperpolarized ΔΨm can induce the accumulation of damaged mitochondria and dysregulate the influx of Ca^2+^ to mitochondria, leading to excessive reactive oxygen species production, which is one of the main hallmarks of various neurodegenerative diseases, including AD [38]. Interestingly, restoring mitochondrial function through various strategies, including antioxidants, has been suggested to be a potential target to delay the progression of AD [39]. To our knowledge, no prior study has reported a positive effect from OXT in improving and reversing the cytotoxic effect of any substance in ΔΨM. Therefore, we believe that our results are novel in linking the protective effect of OXT to the decline in ΔΨM. In light of our findings, we suggest that OXT protects PC12 cells against Aβ-induced reduction in mitochondrial membrane potential.

### 3.4. Investigating Whether the Protective Effect of OXT on PC-12 Cells Against Aβ-Induced Cytotoxicity Involves Modulation of the MAPK and Mitochondrial Apoptosis Pathways

#### 3.4.1. OXT Promotes Cell Proliferation of PC12 Cells by Activation of the ERK1/2 MAPK Signaling Pathway

Our findings indicate that Aβ causes a minimal reduction in the pERK1/2 signaling pathway when compared to the control group. In contrast, the groups treated with oxytocin (OXT) exhibited a significant upregulation of the pERK1/2 signaling pathway relative to both the Aβ-only group and the control group (Figure 5). Previous research has demonstrated that OXT promotes the proliferation of the glioblastoma cell line U-87MG through activation of pERK1/2 protein expression [40]. Interestingly, the effect of OXT has been confirmed in various diseases, including myocardial ischemia-reperfusion injury and Parkinson’s disease [26,27]. Our findings showed that OXT upregulated the pERK1/2 signaling pathway, which may explain the increase in counted cell numbers observed in the TB assay results.

#### 3.4.2. OXT Protects PC12 Cells Against Apoptosis Mediated by Aβ Through Modulation of Mitochondrial Apoptosis Pathway

We demonstrated that Aβ decreases Bcl-2. On the other hand, OXT-treated groups showed a non-significant elevation of Bcl-2 compared with the group treated with Aβ alone (Figure 6). Many studies have associated the reduction in Bcl-2 expression with the cytotoxic effects of different oxidative stressors and pro-apoptotic agents [41,42,43]. Consistent with our findings, a previous study reported that OXT upregulated Bcl-2 expression in a mouse stroke model [44]. In our study, OXT elevated the levels of Bcl-2, an anti-apoptotic mediator, but the effect was not significant. Further research is needed to fully understand the role of Bcl-2 in apoptosis and to develop new therapies targeting the Bcl-2 pathway.

Additionally, our results revealed that Aβ significantly elevates the pro-apoptotic BAX protein. On the other hand, OXT-treated groups showed a significant reduction of BAX protein expression compared with the group treated with Aβ alone (Figure 7). Moreover, a prior study that supports our findings reported that OXT protects PC12 cells from apoptosis by decreasing the expression of pro-apoptotic genes such as Bax [45]. According to our findings and reported studies, OXT can protect PC12 cells against Aβ by downregulating BAX expression, a key apoptosis regulator.

We also determined the BAX and Bcl-2 ratios to assess the apoptotic status of cells and identify potential therapeutic targets. Our results show that Aβ elevates the BAX and Bcl-2 ratio compared with the control. On the other hand, OXT-treated groups showed a reduction of BAX and Bcl-2 ratio compared with the group treated with Aβ alone (Figure 8). A previous study found that dysregulation in Bcl-2 proteins is associated with AD. Specifically, upregulation in BAX protein and downregulation of Bcl-2 protein [21,46]. Based on our findings and previous research, OXT can protect PC12 cells against Aβ by downregulating the BAX/Bcl-2 ratio.

Moreover, our result demonstrated that OXT protects PC12 cells against Aβ-induced modulation of the mitochondrial apoptosis pathway. In our study, we have shown that Aβ elevates the apoptosis executor caspase-3 significantly compared with the control. On the other hand, OXT-treated groups showed a significant reduction of pro-apoptotic caspase-3 protein expression compared with the group treated with Aβ alone (Figure 9). Moreover, a study published in 2009 showed that OXT protected PC12 cells from apoptosis induced by staurosporine by inhibiting caspase-3 activation [12]. Based on our findings and reported studies, OXT has a protective effect on PC12 cells against Aβ by inhibiting the activation of caspase-3.

This research highlights the molecular effects of OXT in mitigating Aβ-induced cytotoxicity through various methodologies. The findings suggest that OXT exhibits mitochondrial protection, antioxidant properties, anti-apoptotic effects, and promotes cell proliferation, which could pave the way for new therapeutic approaches for AD. Indeed, recent clinical trials indicate that intranasal administration of OXT may serve as a promising treatment for various neurological disorders, including dementia and autism [47,48].

## 4. Materials and Methods

### 4.1. Instrumentation

All instruments required for this in vitro study were available in the laboratories of the pharmacology department of King Saud University’s College of Pharmacy.

### 4.2. Reagents

Dulbecco’s modified Eagle’s medium (DMEM) (Gibco, Grand Island, NY, USA), fetal bovine serum (FBS) (Gibco), 100X penicillin/streptomycin (5000 U/mL) and (3-(4,5-dimethylthiazol-2-yl)-2,5-diphenyl tetrazolium bromide (MTT), a Mitochondrial Membrane Potential Assay Kit (JC1), and a ROS measuring kit (2′,7′-dichlorodihydrofluorescein diacetate (H2DCFDA) were purchased from MedChemExpress (Monmouth Junction, NJ, USA). Lastly, phosphorylated ERK1/2, caspase-3, Bcl-2, BAX, GAPDH, and beta-actin antibodies were purchased from ABclonal Technologies (Woburn, MA, USA).

### 4.3. Aβ25–35 Preparation

An Aβ25–35 stock solution was prepared by dissolving Aβ25–35 in double-distilled water to obtain a 1 mM stock solution. To induce Aβ25–35 aggregation (toxic form of Aβ), the solution was incubated at 37 °C for five days, and the aliquots were stored in Eppendorf tubes at −80 °C until use.

### 4.4. OXT Preparation

The OXT stock solution was prepared by dissolving OXT in double-distilled water to obtain a 1 mM stock solution. To avoid refreezing, 10 µL aliquots were stored in each Eppendorf tube at −80 °C until use. The selected concentrations of OXT (10, 100, 1000 nM) are consistent with our established research that has effectively employed similar concentrations of oxytocin to produce significant biological effects in neuronal cells [49,50]. Additionally, we performed a dose–response analysis to confirm that these concentrations do not have any detrimental effects on our study model.

### 4.5. Experimental Groups

PC12 cells were divided into five groups: one control group and four experimental groups comprising OXT pre-treated groups and an Aβ25–35 treated group (Table 1). The cells were cultured in DMEM with 10% FBS and 1% penicillin/streptomycin. The OXT pre-treated groups were exposed to different OXT concentrations before Aβ exposure. We used 96-well plates for the MTT, H2DCFDA–cellular ROS, and JC-1–mitochondrial membrane potential assays and 6-well plates for the Trypan Blue (TB) staining assay. Finally, a T25 flask was used for Western blotting (WB).

### 4.6. Cell Culture

Rat Pheochromocytoma PC-12 cells (ATCC CRL-1721, Passages 5–9; the American Type Culture Collection, Manassas, VA, USA) are widely recognized as a neuron-like model for investigating neurological, neurodevelopmental, and neurodegenerative disorders due to their neuronal-like properties, responsiveness to oxidative stress, and ease of handling [51,52]. These cells were cultured at a density of approximately 1 × 10^6^ cells/mL. The cells were grown in Dulbecco’s modified Eagle’s medium (1×; Gibco^®^, Grand Island, NY, USA) supplemented with 10% fetal bovine serum (FBS, South American origin; Gibco^®^) and 1% streptomycin/penicillin (100 μg/mL and 100 Units/mL; Gibco^®^). They were maintained in a humidified incubator with 5% CO_2_ at 37 °C. Confluent cells from Passages 5–15 were used for subsequent experiments. Here, 96-well plates were used for the cytotoxicity (MTT) (1 × 10^4^ cells/well, 24 h), reactive oxygen species (ROS) (3 × 10^4^ cells/well, 4 h), and mitochondrial membrane potential (JC-1) (2 × 10^4^ cells/well, 24 h) assays. In contrast, 6-well plates were used for the TB and WB assays (1 × 10^6^ cells/well, 24 h). OXT and Aβ treatment was performed in a full culture medium containing FBS and streptomycin/penicillin. The medium was changed every 48 h until the cells reached approximately 80–90% confluence.

### 4.7. Cell Proliferation and Viability Experiments

#### 4.7.1. 3-(4,5-Dimethylthiazol-2-yl)-2,5-diphenyltetrazolium Bromide (MTT) Assay

The MTT assay was used to measure cell viability after exposure to OXT and to identify any protective effect against Aβ cytotoxicity. Briefly, cells were cultured, harvested, and trypsinized as previously described. They were then seeded at 1 × 10^4^ cells/well (in 100 μL of full medium) in 96-well plates. The plates were incubated for 24 h. Viability was evaluated in cells pre-incubated in the presence of varying concentrations of OXT (10, 100, and 1000 nM) for 2 h and then exposed to Aβ for 48 h. At the end of the experiment, 10 μL MTT reagent (5 mg/mL MTT in phosphate-buffered saline, PBS) was added and allowed to stand for 1 h. Next, 100 μL DMSO was added, and the mixture was shaken for 5 min. Finally, absorbance was measured at 570 nm using a microplate reader.

#### 4.7.2. Trypan Blue Staining Assay

Trypan blue (TB) is an azo dye that quantifies live cells and excludes nonviable cells. Viable cells have an intact cell membrane; thus, TB cannot penetrate the cell membrane or enter the cytoplasm. On the other hand, TB passes through the cell membrane and enters the cytoplasm of the nonviable cells. Under microscopic analysis, only nonviable cells are stained blue. Briefly, 3 × 10^5^ cells were seeded and incubated in 6-well plates for 24 h. Then, viable cells and cell proliferation were evaluated in cells pre-incubated in the presence of varying concentrations of OXT (10, 100, and 1000 nM) for 2 h and then exposed to Aβ for 48 h. Next, we counted the total number of cells in four squares of the hemocytometer. After obtaining the cell counts from these squares, we averaged the numbers and multiplied the result by the objective factor of 10,000. Data are expressed as number of viable cells per mL, normalized to the control group.

### 4.8. Measurement of ROS Level

To assess the protective effect of OXT against Aβ-induced ROS generation, we utilized an H2DCFDA–Cellular ROS Assay Kit, using the cell-permeant reagent 2′,7′–dichlorofluorescin diacetate. H2DCFDA is a fluorescent dye that measures the activities of hydroxyl, peroxyl, and other ROS within the cells. Briefly, cells were cultured, harvested, and trypsinized as previously described. They were then seeded at 3 × 10^4^ cells/well (in 100 μL of full medium) in 96-well plates. The plates were incubated for 24 h. ROS generation was evaluated in cells pre-incubated in the presence of varying concentrations of OXT (10, 100, and 1000 nM) for 2 h and then exposed to Aβ for 2 h. Subsequently, H2DCFDA (25 μM) was added to cells and incubated for 30 min. At the end of the experiment, the DCF is a highly fluorescent compound with excitation and emission wavelengths of 485 and 529 nm, respectively, measured using a microplate reader.

### 4.9. Measurement of Mitochondrial Membrane Potential

To assess the protective effect of OXT against Aβ-induced mitochondrial dysfunction, we utilized a Mitochondrial Membrane Potential Assay Kit (JC-1) containing tetra ethyl benzimidazolyl carbocyanine iodide. JC-1 dye, a monomer accumulating in mitochondria, is an indicator for ΔΨm in non-apoptotic cells. Briefly, cells were cultured at a density of 2 × 10^4^ cells/well in 96-well plates and allowed to stabilize for 24 h. They were then pre-incubated with different concentrations of OXT (10, 100, and 1000 nM) for 2 h and then exposed to Aβ for 48 h. Subsequently, cells were incubated with 2 μM JC-1 reagent for 15 min. The effect of different treatments on the mitochondrial membrane potential was evaluated according to the manufacturer’s instructions (Table 2). Finally, the red/green ratio for all the experimental groups was calculated and graphed.

### 4.10. Western Blotting

WB was performed to investigate whether OXT mediated its protective effect against Aβ by modulating the mitochondrial apoptosis and MAPK pathways. The sample preparation: After incubating the cells under optimal conditions, they were washed once with 0.1% Tween 20 in Tris-buffered saline (TBST). Following this, 300 μL of lysis buffer cocktail (comprising lysis buffer and protease inhibitors) was added to each T25 flask, and the mixture was incubated on ice for 20 min. The cells were then harvested and transferred into 1.5 mL Eppendorf tubes. Using a Beckman refrigerated centrifuge, the cells were centrifuged at 1000 rpm for 5 min at 20 °C, and the supernatant was carefully collected into new 1.5 mL Eppendorf tubes. Protein concentration was subsequently measured using a BCA protein assay kit from Thermo Fisher Scientific (Waltham, MA, USA; Cat# 23227). Concerning the WB procedure, we loaded 15 µg of protein into each well of the gel. The solubilized proteins were then separated using 12% SDS-PAGE, followed by transfer to PVDF membranes. The membranes were blocked for 1 h at room temperature (25 °C) with a solution of 5% non-fat dry milk in TBST on a shaker. After blocking, the membranes were washed three times with TBST. Next, they were incubated overnight at 4 °C with specific antibodies targeting the proteins of interest: Bcl-2, BAX, caspase-3, phospho-ERK1/2, and β-actin (ABclonal Technology, Woburn, MA, USA; dilution 1:1000) while gently rocking. The following day, the membranes underwent another three washes with TBST before being incubated for 1 h at room temperature with HRP-conjugated goat anti-rabbit secondary antibodies (ABclonal Technology, USA; dilution 1:5000). Subsequently, the membranes were washed three times with TBST again, and the immunoreactive bands were detected and visualized using an enhanced chemiluminescence (ECL) system (ABclonal Technology, USA). The raw data obtained using a Bio-Rad gel imaging system were quantified using ImageJ software 1.8 from the National Institute of Health (available at https://imagej.nih.gov/ij/, accessed on 6 June 2024).

### 4.11. Statistical Analysis

Each experiment was conducted in triplicates. GraphPad Prism version 8 (GraphPad Software, Inc., La Jolla, CA, USA) was used for the statistical analyses and making of graphs. The data are presented as mean ± standard error of the mean. A one-way analysis of variance (ANOVA) was used to estimate statistical differences between groups, followed by Tukey’s post-hoc test. A *p* < 0.05 was considered statistically significant.

## 5. Conclusions

**Figure 10:**
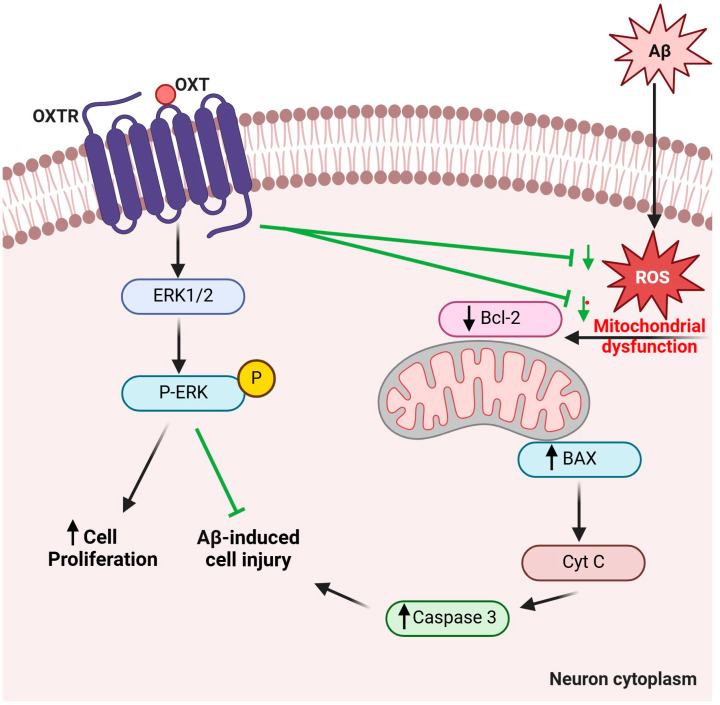
Proposed mechanism for oxytocin protective effects on β-amyloid-induced neurotoxicity. B-cell leukemia/lymphoma 2 protein (Bcl-2); Bcl-2–associated X protein (BAX); Beta-amyloid (Aβ); cytochrome complex (Cyt C); Extracellular signal-regulated kinases (ERKs); Oxytocin (OXT); Oxytocin receptor (OXTR); Phosphate (P); Reactive oxygen species (ROS). The figure was created with BioRender.com.

Our findings revealed that OXT protects PC12 cells against Aβ-induced neurotoxicity. Furthermore, OXT has an antioxidant effect against Aβ-induced oxidative stress. Additionally, OXT protected the cells against the Aβ25-35 cytotoxic effect by diminishing the level BAX/BCL-2 ratio and reducing the expression of pro-apoptotic proteins caspase-3. Interestingly, our study observed that OXT could reverse the impact of Aβ25-35 on the mitochondrial membrane potential. Additionally, OXT led to the upregulation of pERK1/2, which might explain the increase in cell number in the TB assay. Taken together, our data have proposed the neuroprotective mechanism of OXT against Aβ-induced neurotoxicity, as presented in Figure 10. Additional research is required to investigate further protective mechanisms of oxytocin against Aβ-induced neurotoxicity. Moreover, it is essential to conduct additional studies to explore the effects of OXT on neuroplasticity and connectivity within the context of AD. Furthermore, investigations into the behavioral impact of OXT on Alzheimer’s models are necessary to establish a correlation between the molecular effects of OXT and the behavioral changes it may induce in these models. Further studies are needed to address whether these effects of OXT against Aβ are mediated through extracellular interaction between the two or through direct activation of OXTR by OXT. The findings of our study may help clarify the relationships among OXT, neurological disorders, and AD, as well as the protective mechanism of OXT and the clinical benefits of its mediation in AD. Ultimately, these findings provide a glimmer of hope and open a novel path for AD treatment.

## Figures and Tables

**Table 1:** Experimental Groups.

Group 1	Group 2	Group 3	Group 4	Group 5
Control	20 μM Aβ for 48 h	10 nM OXT for 2 h + 20 μM Aβ for 48 h	100 nM OXT for 2 h + 20 μM Aβ for 48 h	1000 nM OXT for 2 h + 20 μM Aβ for 48 h

**Table 2:** Wavelengths for the red oligomers and green monomers of JC-1 reagent.

RED (Oligomers)	GREEN (Monomers)
Excitation/530 nm	Excitation/485 nm
Emission/590 nm	Emission/528 nm

## Data Availability

All data presented in this study are available upon reasonable request from the corresponding authors.
